# Blood Cell Membrane Fluidity and Intracellular Ca^2+^ Changes in Antiretroviral-Naïve and -Treated HIV-1–Infected Patients

**DOI:** 10.1100/tsw.2010.34

**Published:** 2010-02-19

**Authors:** Nuno C. Santos, J. Martins e Silva, Teresa Freitas, M. Doroana, N. Duarte, L. Tavares, F. Antunes, Carlota Saldanha

**Affiliations:** ^1^ Instituto de Medicina Molecular, Faculdade de Medicina Universidade de Lisboa, Lisboa, Portugal; ^2^ Clinica Universitária de Doenças Infecciosas, Faculdade de Medicina Universidade de Lisboa, Lisboa, Portugal; ^3^ Serviço de Infecciologia, Hospital de Santa Maria, Centro Hospitalar Lisboa Norte, Lisboa, Portugal

**Keywords:** HIV-1, lymphocyte, erythrocyte, membrane fluidity, calcium

## Abstract

We previously showed that lymphocytes and erythrocytes of HIV-1–infected patients, prior to antiretroviral therapy, presented significant changes in intracellular calcium concentration ([Ca^2+^]_int_) and membrane fluidity. The present study evaluates the same parameters after response to highly active antiretroviral therapy (HAART). Blood samples were collected from patients prior to and after antiretroviral therapy, and from control subjects. Membrane fluidity and [Ca^2+^]_int_ were assessed by fluorescence spectroscopy measurements, using three different probes: TMA-DPH and DPH for membrane fluidity, and fura-2 for Ca^2+^. When compared with the control group, both untreated and treated patients presented increased lymphocyte [Ca^2+^]_int_ and decreased lymphocyte membrane fluidity, without significant differences between the two groups of patients. On the contrary, the therapy reversed the membrane fluidity variations observed in erythrocytes. The decreased erythrocyte [Ca^2+^]_int_ of untreated patients was not reversed by HAART. AIDS patients present changes in lymphocyte (mostly noninfected) and erythrocyte properties, partially reversed by HAART, consistent with a process of facilitated propagation of the infection to new cells, stimulation of virion production, and maintenance of a reservoir of erythrocyte-bound infectious virus. These observations can be related with the action of the HIV Nef protein in the cell's proteins and lipid composition, as well as with the recently observed cell infection by HIV-1 via endocytosis.

## BACKGROUND

Despite the success of highly active antiretroviral therapy (HAART), it is still unable to eliminate human immunodeficiency virus type 1 (HIV-1) from several cellular and anatomical reservoirs. HIV-1 is not able to replicate in erythrocytes. Nevertheless, infectious virus can be found bound to erythrocyte membranes in the vast majority of HIV-infected patients, creating a reservoir of infective virus and, simultaneously, using them as a "shuttle" to circulate within the organism[1]. When compared with an erythrocyte, there is a larger probability of finding virus attached to a single leukocyte. However, the larger number of erythrocytes in the blood makes them a major reservoir of infectious virus. The lipid composition of the HIV viral membrane (of cellular origin) is similar to the composition of erythrocyte membranes, with a cholesterol-to-phospholipid ratio considerably higher than that observed in other cells of healthy subjects[2]. A recent publication about the HIV-1 lipidome[3] demonstrated that this viral lipid composition results from the cell membrane lipid microdomains richer in cholesterol and sphingolipids, termed lipid rafts, which are preferentially incorporated in the viral membrane during the process of assembly and release from the host cell[4]. Lipid rafts are also involved in the entry of HIV into a target cell (such as T-lymphocytes), mediated by the viral membrane glycoprotein complex formed by gp41 and gp120, which interact with CD4 and a coreceptor (usually CCR5 or CXCR4)[5]. Adding to their particular lipid composition, lipid rafts are also enriched in glycosylphosphatidylinositol (GPI)-anchored proteins, such as acetylcholinesterase (AChE), which can be used as a marker of membrane integrity[6].

After CXCR4 binding to SDF-1α (its physiological ligand), the internalization of the receptor is mediated by $Ca^{2+}$ stimulation[7]. Studies with HIV-infected cultured T-lymphocytic cells have shown that the mobilization of calcium ions from intracellular storage pools (elevating cytosol $Ca^{2+}$ concentration) is a key component for cell activation, a process that could stimulate virus replication[8]. Subsequent studies in cell culture indicated that this calcium signaling, involved in the control of HIV Tat protein activity[9], is modulated by gp120 and chemokines through CCR5 and CXCR4 stimulation[10], and by HIV Nef protein[11]. Nef also induces an increase in cholesterol biosynthesis and changes in its transport[12,13,14,15].

## OBJECTIVES

In a previous paper[6], we showed that lymphocytes and erythrocytes of HIV-1–infected patients, prior to their engagement in antiretroviral therapy, presented significant changes in membrane fluidity, intracellular calcium concentration ($[Ca^{2+}]_{int}$), and AChE activity when compared with a control group of healthy subjects. The present study evaluates the same parameters after response to HAART. It is important to bear in mind that this study does not intend to evaluate these parameters in infected cells. It is conducted with cells from infected patients, but most of these cells are not infected by the virus.

## STUDY DESIGN

Blood samples were collected with heparin (10 U/ml), with previous informed consent, from patients prior to (n = 39–55) and after (n = 39–44) response to antiretroviral therapy, and from healthy subjects (n = 39–56) forming a control group with similar characteristics. A decrease of the viral load to values below 1000 copies/ml was used as criterion for considering that a patient presents a marked response to HAART. All these patients showed $CD4^+$ counts above $300/mm^3$. The laboratorial methods were previously described[6]. Briefly, membrane fluidity and $[Ca^{2+}]_{int}$ were assessed by fluorescence spectroscopy measurements, using three different probes: trimethylamino-diphenyl-hexatriene (TMA-DPH) and diphenyl-hexatriene (DPH) for membrane fluidity (fluorescence anisotropy measurements), and fura-2 acetoxymethyl ester for $Ca^{2+}$. DPH reports the membrane fluidity in the interior of the membrane, at the level of the acyl chains, and TMA-DPH the fluidity closer to the lipid/water interface. AChE activity was measured by a colorimetric method. Statistical analyses were carried out using two-tailed unpaired samples t-tests and Pearson correlation calculations.

## RESULTS

The values determined for the lymphocyte and erythrocyte parameters under evaluation, obtained for the three groups studied, are presented in Fig. 1 together with the statistically significant variations obtained ($p < 0.05$). When compared with the control group, both untreated and treated patients presented a statistically significant increase in lymphocyte $[Ca^{2+}]_{int}$ and decreased lymphocyte membrane fluidity (a higher fluorescence anisotropy value indicates a lower membrane fluidity). There are no statistically significant differences between the two groups of patients, either with TMA-DPH or with DPH. On the contrary, the therapy reversed the membrane fluidity variations observed in erythrocytes, reaching anisotropy values identical to those obtained for the control group. The decreased erythrocyte $[Ca^{2+}]_{int}$ of untreated patients was not reversed by HAART. Regarding AChE activity, the decrease observed for the lymphocytes of antiretroviral-naïve patients was reversed by the therapy. A therapy-associated increase of the AChE activity values was also observed for erythrocytes, where there were no significant differences between the untreated patients and the control group.

## DISCUSSION

Decreased lymphocyte membrane fluidity can be mainly due to the HIV-1 infection–induced alterations in the biomembrane lipid composition; namely, a higher cholesterol-to-phospholipid ratio[3], leading to a membrane-ordering effect. This can result in alterations of the membrane heterogeneous distribution of components (lipid microdomains or lipid rafts), considered to play an important role on HIV entrance in a target cell, and on HIV assembly and release. The less-fluid membrane can be related with the increase of the fraction of the membrane surface covered by rafts and/or with the formation of larger rafts. These events can be related with the formation of an “activated state” by the clustering of several rafts to form a larger platform where the several CD4 and chemokine receptors necessary for HIV binding to the target cell and membrane fusion can be associated. This hypothesis is in accordance with the several models proposed for the general mechanism of lipid rafts action. The relevance of the increased cholesterol content and decreased fluidity of the lymphocyte membranes of HIV-infected patients can also be related with the formation of nonlamellar, highly curved, stalk intermediates (necessary to form a local membrane bend important for membrane fusion), and with the modulation of the activity of several membrane proteins, such as CCR5 and CXCR4[16].

Taking into consideration solely the results obtained for the patients prior to therapy, the decrease in lymphocyte AChE enzyme activity could be explained by the changes in membrane composition and/or lipid microdomain organization (AChE, as other GPI-anchored proteins, locates preferentially in lipid rafts). A higher fraction of membrane area covered by rafts, leading to a lower average surface concentration of GPI-anchored proteins, could lead to a decreased AChE activity[6]. However, this hypothesis is not sustained by the results obtained after therapy, since the increase in lymphocyte AChE activity after treatment occurs without statistically significant changes in membrane fluidity.

It should be stressed that the observations above do not refer exclusively to infected lymphocytes. The measurements result in an averaging of the studied cell population. Considering the low percentage of infected $CD4^+$ T-lymphocytes among the lymphocytes isolated from an HIV-infected patient blood sample, it must be reasoned that the observations result mainly from noninfected cells. Thus, the modification of the parameters referred, instead of a consequence of cell infection, can result from a preconditioning of the noninfected lymphocytes (triggered by patient infection) in order to facilitate the propagation of the infection to new $CD4^+$ T-lymphocytes. Consequently, this may increase the velocity of new virion production. Based on our observations, these purposes are achieved by modulating lipid composition and lipid raft organization (facilitating the entrance of the virus in the cell and the release of new virus), and by increasing the $[Ca^{2+}]_{int}$ (inducing cell activation and stimulating virus replication). We can speculate that the mechanism underlying the observed changes can be by the action of the viral protein Nef. It has been quantitatively demonstrated that Nef alters not only the proteins, but also the lipid

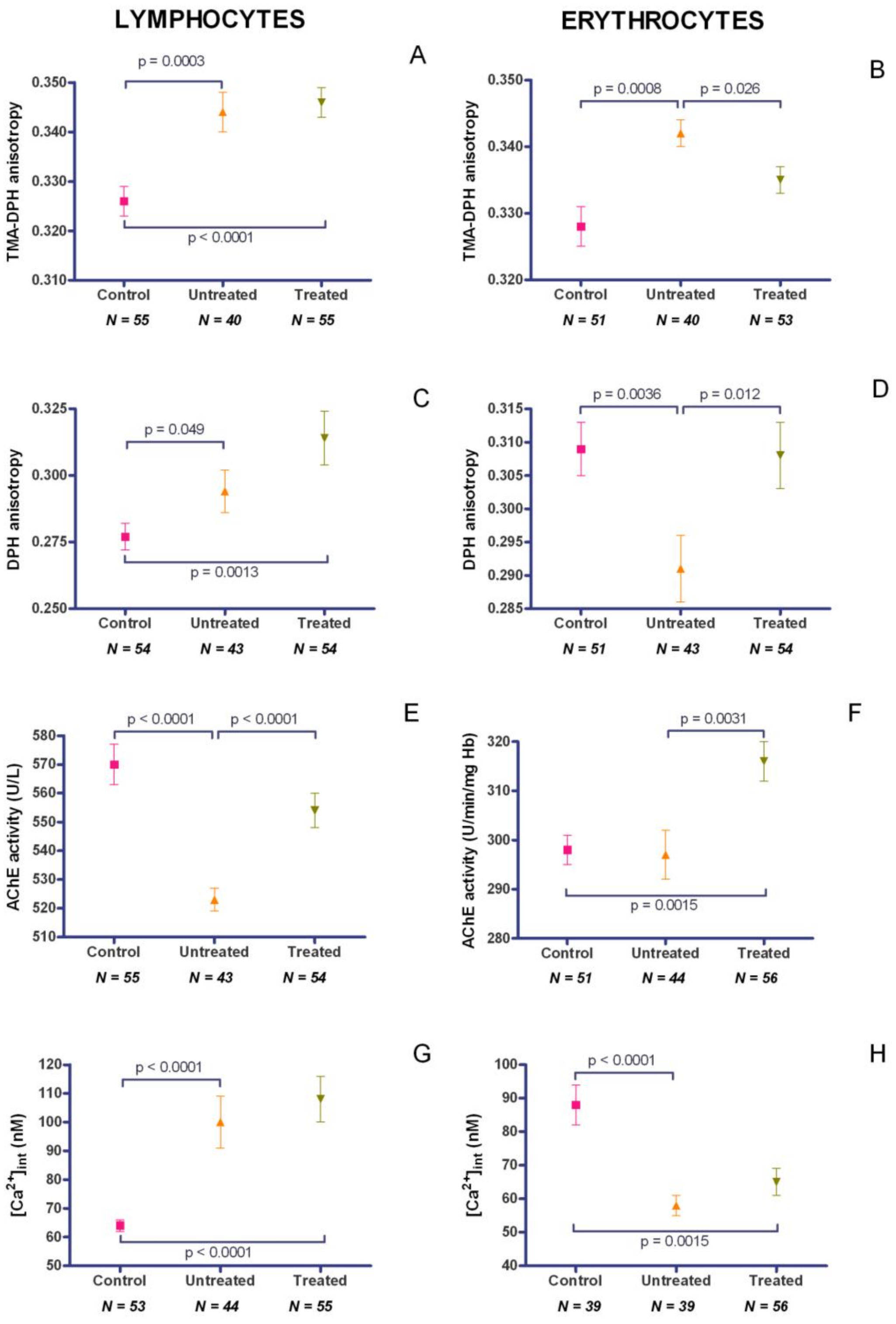

**FIGURE 1.** Values of TMA-DPH anisotropy (A and B), DPH anisotropy (C and D), AChE enzyme activity (E and F), and $[Ca^{2+}]_{int}$ (G and H) determined for the lymphocytes and erythrocytes obtained for the three studied groups: healthy subjects (control), antiretroviral treatment–naïve HIV-1–infected patients and antiretroviral-treated patients. Data are presented as mean ± standard error. The *p* values obtained by the comparison of two groups using two-tailed unpaired samples t tests are presented whenever the variation is statistically significant ($p < 0.05$). All the other pairings yielded nonsignificant variations.

composition of HIV target cells[15]. The decreased cell membrane fluidities observed in the present study can be the direct result from the Nef-induced increase in cholesterol biosynthesis and conditioning of its transport patterns[12,13,14,], eventually even in the noninfected cells of HIV-1–infected patients.

In a recent paper[17], Miyauchi et al. reported evidences of the entrance of HIV-1 into a target cell through an endocytic pathway. This is contrary to the commonly accepted view of an entrance by a membrane fusion process occurring directly at the cell membrane. If that is the case, the infection of an individual would lead to a change in the lipid composition and ordering of the noninfected lymphocytes, leading to a facilitated and faster receptor-mediated endocytosis.

The results obtained with erythrocytes from HIV-infected patients show that the membrane-ordering effects observed near the lipid/water interface (with TMA-DPH) are identical to those observed for lymphocytes. However, the changes in lipid organization of the hydrophobic region of the membrane, probed by DPH, show a different trend. These observations seem in agreement with a process that leads to an increase in erythrocyte-HIV binding, without the need to increase membrane fusion (necessary in the lymphocyte-HIV interaction, but not for erythrocytes). The opposite $[Ca^{2+}]_{int}$ variation trends observed in lymphocytes and erythrocytes can be related with an overall depletion of calcium in noninfectable cells and/or to the absence of intracellular compartments in erythrocytes.

It is worthy of notice that most of the treatment effects occur on the erythrocyte parameters. Regarding the restoration of erythrocyte membrane fluidity after treatment, one possibility is that the decrease of the number of viruses bound to erythrocyte membranes, as a consequence of the decrease in viral load due to the treatment, could restore membrane properties.

Considering that AIDS patients present changes in lymphocyte (mostly noninfected) and erythrocyte properties consistent with a process of facilitated propagation of the infection to new cells, stimulation of virion production, and maintenance of a reservoir of erythrocyte-bound infectious virus, the HAART-associated reversion of some of the variations to values identical to those observed for healthy subjects indicates an at least partial inactivation by the therapy of this process of facilitated propagation of the infection to new cells.

## ACKNOWLEDGMENTS

The authors thank Dr. Graça Lourenço and Dr. Ricardo Teixeira (Serviço de Hematologia, Hospital de Santa Maria) for the flow cytometry measurements and to Mrs. Carmo Fernandes (Faculdade de Medicina da Universidade de Lisboa) for technical assistance. This project was supported by the *Coordenação Nacional para a Infecção VIH/SIDA – Alto Comissariado da Saúde* of the Portuguese Ministry of Health.
